# Social Processes That Can Facilitate and Sustain Individual Self-Management for People with Chronic Conditions

**DOI:** 10.1155/2012/282671

**Published:** 2012-11-22

**Authors:** Elizabeth Kendall, Michele M. Foster, Carolyn Ehrlich, Wendy Chaboyer

**Affiliations:** ^1^Centre for National Research on Disability and Rehabilitation Medicine, Griffith Health Institute, Griffith University, Logan Campus, Meadowbrook, QLD 4131, Australia; ^2^School of Social Work & Human Services, The University of Queensland, St. Lucia, QLD 4072, Australia; ^3^NHMRC Centre of Research Excellence in Nursing Interventions for Hospitalised Patients, Research Centre for Clinical and Community Practice Innovation and Griffith Health Institute, Griffith University, Gold Coast Campus, Southport, QLD 4215, Australia

## Abstract

Recent shifts in health policy direction in several countries have, on the whole, translated into self-management initiatives in the hope that this approach will address the growing impact of chronic disease. Dominant approaches to self-management tend to reinforce the current medical model of chronic disease and fail to adequately address the social factors that impact on the lives of people with chronic conditions. As part of a larger study focused on outcomes following a chronic disease, this paper explores the processes by which a chronic disease self-management (CDSM) course impacted on participants. Five focus groups were conducted with participants and peer leaders of the course in both urban and rural regions of Queensland, Australia. The findings suggested that outcomes following CDSM courses depended on the complex interplay of four social factors, namely, social engagement, the development of a collective identity, the process of building collaborative coping capacity, and the establishment of exchange relationships. This study highlights the need for an approach to self-management that actively engages consumers in social relationships and addresses the context within which their lives (and diseases) are enacted. This approach extends beyond the psychoeducational skills-based approach to self-management into a more ecological model for disease prevention.

## 1. Introduction

With a rapid rise in the prevalence of chronic conditions and the ensuing demand placed on health services, the sustainability of most health care systems around the globe has been threatened [1]. During the last decade, the strategy of choice has been to focus on promoting healthy lifestyles and choices [2, 3], the most common method of which has been to promote self-management through the delivery of psychoeducational group programs. This approach has now become an integral component of the Australian and UK healthcare systems. Although there are multiple approaches to the promotion of self-management, the most common approach has been the Lorig [4] model of chronic disease self-management (CDSM). This model is a standardized course delivered over 6 weekly sessions of approximately 2hours each week. Courses are delivered in community settings and usually facilitated by two trained peer leaders using a highly structured course protocol. Course content introduces participants to a range of topics pertaining to health and well-being (e.g., healthy eating, exercise, relaxation). The process emphasizes group interaction and support and reinforces solution-focused behaviors (e.g., problem solving, goal setting, communication with healthcare team and family) aimed at assisting individuals to actively manage the impact of chronic conditions on all domains of their life (e.g., emotional, physical, and social well-being).

 Within this approach, health professionals are primarily responsible for the medical management of the disease or chronic condition, and the individual is responsible for the day-to-day management of his or her condition. The emphasis is on strengthening individuals' skills and confidence about managing their chronic conditions through supportive group education and improved partnerships between individuals and their health professionals [5]. Self-management remains an individual-level concept framed within a medical model, focused on disease and deficiencies in the person which require education to enable them to comply with health professional advice [6]. In this sense, the CDSM model does not represent a radical shift from traditional approaches to healthcare.

 The purpose of this paper was to identify the way in which participants and leaders of the CDSM course described the mechanisms by which it impacted on them and their health. 

## 2. Method

Five focus groups were conducted during the national implementation and evaluation of the CDSM course in Australia. The purpose of this paper was to examine the way in which the course impacted on health from the perspective of participants (e.g., people who had completed the course within the last six months) and peer leaders (e.g., people with chronic conditions who had run a course for others in the last six months). All eligible leaders and participants who had completed a course in one of the two pilot areas were telephoned and asked to participate in a focus group. Initial contact was made by the organization responsible for the delivery of CDSM training in Queensland, Australia. Those who agreed to participate were then contacted by the research team following approval from the University Research Ethics Committee. 

Care was taken to ensure reasonable representation of male and female participants from a range of differing course locations and people with a range of chronic conditions. However, as expected given the population of participants and leaders, there was a bias towards female participants and an absence of male peer leaders. All participants were over 50 years of age in accordance with the eligibility requirements established by the organization. The constitution of each focus group is shown in Table 1 and the focus group questions are contained in Table 2. 

Focus groups were facilitated by two researchers and were held in the most convenient local building chosen by the leaders of the courses. The focus group discussions were introduced to the participants as having been designed to elicit their perceptions and experiences of the course. Specific prompt questions focused on their awareness and acceptance of self-management as a concept, experiences of the self-management training (where relevant) and course leadership, interactions among participants and followup with health care providers, perceptions of sustainability of self-management, and overall satisfaction with the program. The focus groups were audio-recorded, transcribed verbatim and analyzed using a collaborative multiwave process. 

Two researchers independently coded the transcripts, selecting units of text that contained information about how participants viewed the course and the way in which it had influenced outcomes. Units of text that did not contain any useful information about the course or its influence were discarded (N.B. discarded text usually contained general interactions or comments about benign topics such as the weather, the environment, and personal communications). The units of text selected by these two researchers were compared and discussed to reach agreement about the most important extracts that should be further analysed. Although a few minor pieces of text were discarded as having no meaning for the current study, the two researchers agreed that all other pieces of text should be retained. 

Once this first level of data selection was complete, the reduced dataset was analysed by a third researcher to identify the major themes that existed across all selected extracts. The themes that emerged from this second wave of coding were reexamined by another researcher to determine the extent to which the categorization process was transparent and meaningful. Areas of disagreement were minimal but were addressed through discussion. If text added a useful dimension to several themes, it was used in multiple places. Any text that could not easily be categorized was reviewed. If considered by mutual agreement that the text added nothing new to the analysis, it was discarded. Themes reflected both positive and negative articulations of the concept. 

To validate the findings, wepresented them to a group of peer leaders and trainers as well as national and international experts in the area of CDSM. Feedback indicated that the themes accurately reflected the experience of others in the field. Direct quotes have been replicated verbatim and have been referenced using abbreviations to indicate the source (e.g., U: urban participants, R: rural participants, PL: peer leaders).

## 3. Results

Participants held strong beliefs about the benefits of the course (e.g., knowledge about chronic disease, self-management skills, problem-solving/coping skills, goal setting and decision-making skills). As expected, they reported that their knowledge increased as a result of the course and that this translated into an increased sense of confidence, greater control over their future, and a positive attitude towards their disease. These findings are presented in more detail elsewhere [7].

Participants in this study reported that some potential attendees had elected not to enroll in the course because they disliked group processes. Similarly, some participants failed to complete the course because they had not enjoyed the group format. This conclusion suggests the possibility of a self-selection bias towards those who valued social exchanges. Nevertheless, there was little doubt that those who attended the course attributed their gains to the social context of the course. Specifically, self-management appeared to evolve through, and was situated within, a network of social exchanges and support processes that were facilitated by the course. Indeed, the majority of participants who completed the course discussed social processes more often than course content, indicating the importance of these processes to their evaluation of the course. Participants' level of satisfaction with the social processes of their particular group also seemed to be critical to their overall impression of the course. There was evidence that without this contextual feature of the course, the benefits may have been less meaningful to participants. Further, there was evidence that when social processes were negative, the benefits of the course were jeopardized.

The four major social themes that emerged described the importance of the social context to the success of the CDSM course. These themes included

- social engagement;
- a collective identity;
- collaborative coping capacity;
- exchange relationships.

### 3.1. Social Engagement

An overwhelming theme in the data was the benefit derived purely through social engagement. Participants usually referred to the course as an opportunity for social interaction and described how this interaction addressed the long-term loneliness or social isolation associated with having a chronic condition. 

In most cases, the group provided a friendly context within which people learned about each other's experiences but felt no pressure to divulge personal information. This common experience enhanced the likelihood of supportive friendships emerging, even if only temporarily.

* I found it helpful to mix with people who had similar problems, even though they had different diseases. It was just so supportive (PL).*

*You make friends with people that go through similar pain as you. Each one of us identified with it (R)…It is the best thing that ever happened to me. Because you make friends and we do not see each other all the time but it is just nice to see their faces again (R).*

Having the time and opportunity to socialize with other group members before and after each session was considered to be a valuable aspect of the course for most participants. Their comments indicated that a great deal of satisfaction accompanied these opportunities for social contact.

*So when we first arrived which was always good, if you were there a few minutes early you could have a cup of tea…it was really nice to have a drink and a conversation just for five minutes (U).*

In many instances, the chance for social interaction was a major source of motivation not only to join the CDSM program, but also to continue attending sessions and participate in activities designed to impart information and skills.

*It [course] was the chance of getting out…It does not matter what the group is, it's the social interaction [that matters] (U)…As we went along, we got friends and you know we all joined together (U).*

The value placed on social engagement was demonstrated in the actions of several participants who made the effort to maintain regular contact with other group members once the course had ended.

*We all meet up once a month now and have lunch together and we are going to try and keep it that way (U)…At the follow-up meeting people had actually kept in touch with each other. They seemed to find that very helpful (PL). *

Many participants reported that the CDSM course was a significant opportunity to address social isolation. The course not only provided social opportunities, but, enabled them to reevaluate their own self-isolating behaviors and choices.

*There's a lot of people who do these courses who are very lonely (U)…I'd done like a similar sort of course. I thought well, it's one way of learning more, and um, and meeting people (U).*

*We try…and promote the fact that there is social life ahead for you too, we [people with chronic conditions] have a reluctance to even go outside, to catch a bus. I hated to go down to the letterbox because somebody would see me and I would have to talk (PL).*

*There's that opportunity for social interaction that's important for many people. Because people do tend to feel a bit isolated do not they? Or it's perhaps restricted. It does not matter what group it is. It's the social interaction with it [that matters] (U).*

*To know that there are other people there and there is a social life…encouragement to do something that we needed more than anything else [to meet people] (R).*

In cases where participants' expectations for socialization were not met through the course, the perceived benefits derived by those participants appeared to be reduced, *“I think I was hoping for it to be a little more social for people, like a little more friendly” (U*). Similarly, when participants were dissatisfied with their group, it was often attributed to a lack of social engagement or bonding among participants.

*Nobody was sort of friendly or wanted to [get to know each other]…It was a really mixed group of people…I did not feel, like if you had a “cuppa” afterwards there wasn't much talking going on and they did not talk from the way in from the car park. We went out to lunch the last day but…they really had to be forced into it (U). *

These findings suggest that the benefits of the course which have previously been attributed to cognitive or educational processes may be equally attributed to the simple process of social engagement that was facilitated by the group setting. There was a dual benefit of social engagement in that it motivated participants to initially engage in self-management but also to continue learning.

### 3.2. A Collective Identity

Positive changes in confidence and attitude following the course appeared to be associated with the sense of belonging to a cohesive group of people. The cohesion of the groups provided an immediate opportunity to identify shared concerns, to normalize one's difficulties, to gain a sense of accountability to the group, and to be guided by the norms that had been set by the group. This sense of belonging provided a collective identity that encouraged people to view themselves and their situation differently.

A large number of participants commented on the importance of group composition and dynamics to the success of the course and its benefits, *“I think a lot of it has to do with the people who are in the class” (R).* Participants who felt that their group had lacked cohesion reported that this had impacted negatively on their satisfaction and achievements.

*The class was excellent, the only thing that I thought about it was that I felt a bit out of it—they [other participants] have all got these beautiful homes, beautiful spas and beautiful pools and exercise bikes. The whole works, and I am coming from a rather grotty home and I would have loved to have lived in their circumstances, I felt life could have been a lot easier. But they were sort of, they all knew each other, it was a bit “clicky” in some ways, I felt it…They were all friends, they all knew each other very well and…In comes a couple of outsiders…(U).*

The crucial importance of group membership was summarized by several participants, who pointed out that any group might bring similar benefits if a sense of cohesion could be achieved.

*Any group therapy helps you though…it is just a case of getting together and finding other people…You are not on your own (R)…I'm just one of many people with a problem and by coming together as a group you talk and it gives you another outlook on life. You think you're in that one little square, but…there's other people in that little square too (R). *

For most participants, the fact that they were “…*answerable to somebody”* was an important source of motivation, because of knowing that* “somebody is sharing an interest in you *…*[made you] *…*more inclined to respond” *(*R*). For some participants, however, the pressure of being scrutinized by a group compelled them to offer socially desirable responses during feedback sessions rather than admit that they had not achieved their weekly goals. Thus, the influence of the collective on individual behavior was both positive and negative.

*The lady I took [to the course], on the way I would say, “How did you go with your weekly plan?” and she would tell me, “I did not do anything" and then we would get there and she would say “Oh yes I [completed my action plan]" (U).*

Participants generally agreed that the group norms (e.g., sharing goals and reporting back) meant* “you had that incentive *…*you had to go back and say when you had done it” *(*U*). Participants who had not attended to their course requirements (e.g., goal-setting homework) commented that “…*you really felt you were letting the team down to some extent if you did not at least try” *(*U*). For one person, it was *“like a promise, and you find when you are not there [part of a group] you do not really do it” *(*R*). Indeed, being a member of a cohesive group instilled motivation for most members to achieve their weekly action plans, *“Over the period of time, I think the group helped one another to try to keep with their activity sheet” (R).*

In summary, our findings suggested that the CDSM group context provided an important opportunity for social comparison, normalization, and a sense of belonging. These benefits appeared to be only achievable through a cohesive group where members shared experiences, motivated each other, and provided opportunities for discussion. When members felt they did not belong, or were unable to meet expectations, the outcomes of the course appeared to be less positive. 

### 3.3. Collaborative Coping Capacity

Participants frequently commented on “*the supportiveness of the group, it was very supportive*” (*U*). Most participants were in agreement that, “*when around the table with other people *…*one on one *…*[it was] much easier to cope with your pain*” (*U*). Attendance at the group appeared to be associated with increased coping capacity for many participants. The belief that one's coping efforts were being supported and appreciated by others in the group was an important positive outcome for most participants. 

However, this effect appeared to have broader implications in that coping became a collective response to a public issue rather than a private response to a hidden problem. With this new approach to coping, many participants gained renewed enthusiasm and energy, facilitating their engagement in self-management. Although it was important to participants to develop more confidence to manage independently, they also identified the need for, and importance of, collective management. For many participants, collective spirit and individual confidence appeared to coexist and complement each other.

*Number one [e.g., the most important thing] is better confidence in yourselves [but also] the fact that they're not isolated in their condition and that other people share similar things (R).*

*The shared experience of being with people who have had similar issues has given them [participants in the course] confidence to tackle stuff that they previously wouldn't have done…they are breaking out of the sick role into more lifestyle issues (U).*

Through their shared experience of coping, private pain became a collective experience and was, therefore, perceived as being easier to manage, *“I was not the only one in the community going through pain and disability” *(*R*). The collective environment provided the necessary opportunity to express fears, concerns, and issues in a way that had not been experienced before. This experience profoundly affected participants' connection to the group and their sense of solidarity as they confronted the shared threat of chronic illness.

*I had been to lots of these things [courses] and they left you feeling wrecked…what I found with these meetings is how relaxing they are, how easy it is to gather information. People are given opportunities to be able to speak or express their feelings and where you are given opportunities you are given choices and there is no pressure put on anybody to perform. It is just about people wanting to help somebody else through their daily lives (R).*

The group connection was an important starting point for a collective coping response because group members tended to track each other's coping efforts over time and celebrated the successes as a collective.

*[It is good] to see how we are growing, in ourselves you know. How we are coping with our lives, yes it [the group] is very important (R).*

Conversely, participants described how the presence of negativity in the group impacted on the prevailing collective attitude and had negative consequences for their own psychological well-being and experience, *“A lot of people did their weekly plan [only with] prompting *…*they never did it [alone]”; “they would make excuses” (U).* The lack of motivation in other group members had negative consequences for several participants. *“[It] made you feel depressed”, “oh yes, I did too, I got depressed too” (U). *One participant explained how negativity and lack of motivation in other group members influenced all members of the group:

*Some of the people had given up you know just sort of given up and said, “I just cannot do this” …and you just sort of felt, “Am I going to be like that down the line”? (U). *

In contrast, one participant explained how exposure to unmotivated individuals fortified her determination to cope and successfully manage her condition in future. The collaborative process motivated this participant to resist the negative influence of another participant, identifying that participant as a deviation from the norm and finding motivation to avoid similar outcomes for herself.

*Like I said, once I got out of the group and sort of finished the course, I just sort of kept saying to myself, “There is no way I am going to end up like that, there is no way I am going to end up like that” (U).*

This theme described the importance of coping as both an individual and collective process. Participants reported interacting with each other in complementary ways to facilitate better outcomes for all participants. The coping capacity of the entire group influenced individuals and shaped the strategies they applied beyond the group context.

### 3.4. Exchange Relationships

The process of learning from others, swapping ideas within the group, and sharing information about resources was vital to improvements in confidence, sense of control, and positive attitudes. Essential exchange relationships operated throughout the course, and for some participants, continued after course completion. Participants were inspired not only by their capacity to learn from others, but also by their capacity to share with others. The opportunity to provide information as well as gain information from others was a mutually satisfying activity. This two-way learning process was crucial and encouraged participants to conclude that the course was an important adjunct to the current range of available resources

*Doctors just say go home and look after yourself…whereas if you know there's a group you can go to [the course] and their [other participants'] ideas are so important because one of the persons in that group might have had an illness before and know how to handle situations (PL).*

*If I can swap something that suits me with somebody else and make it a bit of a benefit out of it then that's the idea of these little groups getting together (U).*

All participants reported sharing resources with each other, indicating the universal nature of this exchange function. Most participants appreciated the exchange of ideas and resources among the group members because it enabled new learning to take place for all parties. It encouraged group members to examine their own role in society and feel that they had contributed to the well-being of others.

*You might have a certain problem, but if you start talking to one another, “Oh yes I had that and this is how I got around it”. In other words, it is a swapping of thoughts (R). *

*And so, to know that there are other people there and there is a social life for encouragement …that there is a place for us within the community. Not so much…help because I did not realize I needed help, but I'd like to think that my life is [now] a bit more worthwhile (PL).*

The deliberate creation of dyads who could motivate each other and promote the exchange of ideas was useful to many participants. However, there were examples where this “buddy” system did not work well, because not all participants valued such intimate exchanges with another person.

*Nobody did it [called their buddies] and I felt really silly because I got the attitude when I did ring that I was a sticky nose that I was interfering. I got that impression from them (U).*

*I wouldn't participate in that [buddy system] because I am not that kind of person. I'm not a buddy person like that…I mention that because there might be a few other people like me and do not participate in that. I should imagine it works for a lot of people. But I am afraid I am just not that person (U).*

Indeed, the potential for conflict within dyads was evident. One participant relayed a negative encounter that occurred during a session requiring group members to pair up and discuss negative emotions. This experience highlighted the importance of exchange systems that emerged naturally within the broader group process as opposed to forced dyads that could result in damage to one of the parties if the exchange was not mutual.

*As far as the other people went, we had a major problem with the first or second week, I forget which one. One of the ladies came and she was next to me and I turned around and said to her, “Well would you like to tell me your problems”, because that is what we were meant to do [for the activity], and she attacked me. Really attacked me, as if I wouldn't know what a problem was and she had the worst problems and things. I wouldn't have gone back except that they [leaders] said, “Well she [the woman who had been defensive] is not coming back, she is obviously not right for the course”. So I thought, “Oh well”, I had promised to take [friend] every week so I was forced to go because I had committed myself (U).*

The social rather than interpersonal nature of the group was also highlighted by the fact that participants most commonly reported gaining benefits from processes that engaged the entire group. These activities were viewed as an effective mechanism for social exchange,* “All the work was done on the [white] board and we could all participate” (U).* Participants recognized that practicing new techniques in the group setting, rather than just discussing them, was an important part of the learning process. They noted the values of the immediate performance feedback that could be gained from other participants.

*They were not just actually telling you about it, they got you down [doing the techniques]. It must make a difference if they take you through it (R). *

*We all got to see each other [practice the techniques]…I think there was interest and hoping that we would learn something and, be entertained too (U).*

*You learned something and you were also with a group of people, you know, you weren't just a single person; you were going to learn from others; You exchange experiences, you learn from other peoples' way of coping that you hadn't thought of and sometimes you hear much worse problems than your own too and how the other people coped with them (U). *

This theme revealed an important social exchange function of the course. Instead of relying only on the information provided through the standardized course content, participants sought a two-way exchange of ideas and social comparison with other participants. This process enabled them to find new strategies, resources, and processes that helped them to manage their conditions. They also gained from the opportunity shareing their successes with others. However, this social exchange process differed from the interpersonal support that might be received through a closer relationship with one person.

## 4. Discussion

The central argument developed and presented in this paper is that, far from being an individual concept situated in the private lives of people with chronic conditions, self-management is better understood as a social concept embedded within and facilitated by collective processes and supportive systemic contexts. Over the last decade, increasing emphasis has been placed on the social context within which an individual with a chronic condition is located and the important role of social supports, service infrastructure, and social connections [8]. Despite the importance of individual disease treatment, we have previously drawn attention to the limitations of an individual model of self-management [6]. We have also argued that if inadequate attention is given to the social and environmental factors that can facilitate or inhibit health, self-management efforts may be wasted [9]. 

Our conclusion is further strengthened the key themes that emerged through this analysis of the process by which participants and peer leaders described the impact of the course. Specifically, this study has demonstrated that the social aspect of the group was a crucial factor in the success of the course and that benefits were associated with the interaction of four main social processes. The social context of the course created an environment characterized by collaborative coping, shared learning, and belonging. Most importantly, the course provided a solution to the social isolation that was experienced by many people with chronic conditions. According to participants, these features were linked to the successful outcomes of the course.

This study confirms the raft of evidence that social support is a critical buffer, potentially mitigating the impact of a disabling condition, ameliorating anxiety, and enhancing quality of life [8]. Indeed, there is evidence that high levels of social support are associated with better self-management behaviors [10]. The importance of combining educational and social processes has been found elsewhere [11], suggesting that, although any social gathering might facilitate similar positive outcomes, the course provided the structured interactions that enabled participants to engage in positive ways (e.g., to develop collaborative coping and a collective identity). Choi et al. [12] noted that group members are exposed to two types of influences: (1) discretionary influences that are available to different group members at different times and in different forms as they interact with other group members (e.g., messages of approval, learning, etc.) and (2) ambient influences that are available to all members and pervade the group setting (e.g., group norms, positive climate, shared ideas, etc.). The current study has articulated these different influences, noting the presence of both ambient (e.g., a collective identity and collaborative coping) and discretionary qualities (e.g., social engagement and exchange relationships). 

Despite being delivered in a group setting, the dominant conceptualization of self-management is an individual approach and framed within a medical model. Self-management in this context is defined by three key premises, namely:

- the individual is perceived to be dealing with the consequences of disease;
- the individual is perceived to be deficient in skills such as problem solving, decision making and self-confidence;
- the individual is placed in partnerships with a health professional who takes responsibility for medical management [13].

In contrast to this conceptualization, the current study has suggested that self-management is a social concept and that several important social processes might be able to account for the outcomes achieved through CDSM courses. This analysis has defined a “social” model of self-management that may be more sustainable and relevant than the current individual model of self-management. By giving adequate attention to the social aspects of self-management, it is likely that the utility and meaningfulness of the course could be enhanced for a significant proportion of the population. 

Self-management as a social concept goes beyond individual interventions and even beyond partnerships with health service providers. It may be better conceptualized as a collaborative concept enacted when individuals come together, although not necessarily in a physical place. The act of coming together creates greater capacity to address the “collective” problems associated with chronic disease. The process of self-management seems to be about sharing approaches to common problems, building resources together, encouraging and motivating each other and transforming private pain into collective responses that would never have emerged in an individualized setting. Thus, health professionals may need to refine their understanding of and support for the social processes that contribute to and sustain self-management outcomes.

The process of social self-management that emerged from this study resembles the notion of cultural health capital [14]. According to Shim [14], cultural health capital accrues as one engages in the repeated enactment of health practices (e.g., consuming information, decision making, self-surveillance, etc.). Thus, cultural health capital has a self-generating quality, accumulating over time through interactions with others. This concept is embedded in social processes and is inherently relational. Rather than placing demands on people to become independent and self-directed managers of their own health through education, the notion of self-management as a form of cultural health capital acknowledges that self-management relies on interdependence and builds over time as people engage with new practices and ideas. 

## 5. Conclusions

The findings of this study revealed that responses to disease and ways of self-managing were clearly situated not only in the private lives of individuals, but also in collective processes. Individuals were encouraged and motivated by the social interactions, engagement, and support they received from coparticipants. These findings suggest a dynamic and multidimensional approach to health and well-being which recognizes the role of context and relational aspects of people's environments. Although not surprising, the current study highlights the fact that the dominant interpretation of self-management adopted by many health professionals may be overly simplistic. The focus on skills, resources, and education about health overlooks the importance of building opportunities to enhance one's cultural health capital through positive social interactions. Our study has suggested that there may be sufficient reason for policy makers and professionals to become concerned with activities and interventions that develop supportive social environments and opportunities in addition to their current focus on lifestyle change at the level of the individual. 

However, such a shift will not be easy. Recognition of a social model of self-management will require a fundamental reorientation of professional practice. First and foremost, it will require a shift from the individualistic educational model of self-management towards one based on the application of broad social strategies that can create conditions that foster hope, healing, empowerment, and social connection as well as a positive culture [15]. This shift will require a commitment to new ways of working with clients that reflect the social context within which they function. If health professionals can be encouraged to think about self-management as a form of cultural health capital, accumulated through a vast array of social interactions, they may be able to not only support the social processes that facilitate self-management, but also enact their own role in ways that act as a source of self-management support. The CDSM course appears to be a useful vehicle for facilitating the social processes that emerged from our data. However, it may be possible to promote these social processes more widely within all clinical interactions if a more social view of self-management was propagated. A continued focus on self-management as an individual responsibility that is reliant on the skills and knowledge residing within the individual will encourage health professionals to overlook the social and contextual nature of the concept. It will also enable them to minimize the importance of their own role as a social agent and a facilitator of social processes.

## Figures and Tables

**Table 1:** Focus group participants.

	No. of focus groups	No. of participants	Gender	
			Male	Female
Urban participants	2	16	2	14
Rural participants	2	11	4	7
Peer leaders	1	7	0	7

Total	5	34	6	28

**Table 2:** Focus group prompt questions.

Overall satisfaction with the program	Overall, how satisfied are you with the program?
	What has been the impact (if any) of the program on your life?
Perceptions and experiences of orientation, education, and training	How well were you informed about the program when you first joined?
	What did you know about the program before you commenced?
	What were some of your expectations about the program?
	Overall, how satisfied have you been with the training you received?
	Overall, how satisfied have you been with the postprogram followup?
	What type of support (if any) have you received after program?
	Are there any difficulties you experienced while participating in the program?
	What strategies did you use to overcome these difficulties?
	What kept you coming each week?

Perceived impact of the program	Has the program had an impact on
	the way you manage your condition/s?
	your lifestyle in general?
	How has it changed your lifestyle?
	What are some of the supports/strategies you have used yourself (or are necessary) to make this impact last?
	To what extent did the program leaders answer your questions?
	To what extent do you feel that the program leader gave you adequate information about your condition/s?
	Overall, how would you describe the quality of the program leader

## References

[1] Yach D, Hawkes C, Gould CL, Hofman KJ (2004). The global burden of chronic diseases: overcoming impediments to prevention and control. *Journal of the American Medical Association*.

[2] Gillespie N, Lenz T (2011). Implementation of a tool to modify behaviour in a chronic disease management program. *Advances in Preventive Medicine*.

[3] Singh D How can chronic disease management programmes operate across care settings and providers?. http://www.euro.who.int/__data/assets/pdf_file/0009/75474/E93416.pdf.

[4] Lorig K (1996). Chronic disease self-management: a model for tertiary prevention. *American Behavioral Scientist*.

[5] Lorig KR, Holman HR (2003). Self-management education: history, definition, outcomes, and mechanisms. *Annuals of Behavioural Medicine*.

[6] Kendall E, Rogers A (2007). Extinguishing the social?: state sponsored self-care policy and the Chronic Disease Self-management Programme. *Disability and Society*.

[7] Kendall E, Foster M, Chaboyer W, Gee T, Catalano T (2004). The sharing health care initiative: a better approach to managing
chronic conditions.

[8] Muenchberger H, Kendall E, Han H (2010). Human infrastructure in health: a commentary on networks of supports. *Australian Health Review*.

[9] Kendall E, Ehrlich C, Sunderland N, Muenchberger H, Rushton C (2011). Self-managing versus self-management: reinvigorating the socio-political dimensions of self-management. *Chronic Illness*.

[10] Gallant MP (2003). The influence of social support on chronic illness self-management: a review and directions for research. *Health Education and Behavior*.

[11] Catalano T, Dickson P, Kendall E, Kuipers P, Posner TN (2003). The perceived benefits of the chronic disease self-management program among participants with stroke: a qualitative study. *Australian Journal of Primary Health*.

[12] Choi JN, Price RH, Vinokur AD (2003). Self-efficacy changes in groups: effects of diversity, leadership, and group climate. *Journal of Organizational Behavior*.

[13] Bodenheimer T, Lorig K, Holman H, Grumbach K (2002). Patient self-management of chronic disease in primary care. *Journal of the American Medical Association*.

[14] Shim JK (2010). Cultural health capital: a theoretical approach to understanding health care interactions and the dynamics of unequal treatment. *Journal of Health and Social Behavior*.

[15] Jacobson N, Greenley D (2001). What is recovery? A conceptual model and explication. *Psychiatric Services*.

